# Monitoring of Circulating CAR T Cells: Validation of a Flow Cytometric Assay, Cellular Kinetics, and Phenotype Analysis Following Tisagenlecleucel

**DOI:** 10.3389/fimmu.2022.830773

**Published:** 2022-03-02

**Authors:** Andreas Peinelt, Melanie Bremm, Hermann Kreyenberg, Claudia Cappel, Julia Banisharif-Dehkordi, Stephanie Erben, Eva Rettinger, Andrea Jarisch, Roland Meisel, Paul-Gerhardt Schlegel, Olaf Beck, Gesine Bug, Jan-Henning Klusmann, Thomas Klingebiel, Sabine Huenecke, Peter Bader

**Affiliations:** ^1^Division of Stem Cell Transplantation and Immunology, Department of Children and Adolescents, University Hospital Frankfurt, Frankfurt, Germany; ^2^Department of Children and Adolescents, University Hospital Frankfurt, Frankfurt, Germany; ^3^Division of Pediatric Stem Cell Therapy, Department of Pediatric Oncology, Hematology and Clinical Immunology, Medical Faculty, Heinrich-Heine-University, Duesseldorf, Germany; ^4^Department of Pediatric Hematology and Oncology, University Hospital Würzburg, Würzburg, Germany; ^5^Department of Pediatric Hematology/Oncology, Center for Pediatric and Adolescent Medicine, University Medical Center of the Johannes Gutenberg-University Mainz, Mainz, Germany; ^6^Hematology/Oncology, Department of Internal Medicine, University Hospital Frankfurt, Frankfurt, Germany

**Keywords:** acute lymphoblastic leukemia, chimeric antigen receptor (CAR), immunotherapy, flow cytometry, immune monitoring

## Abstract

Chimeric antigen receptor (CAR) T cell therapy is a potent new treatment option for relapsed or refractory hematologic malignancies. As the monitoring of CAR T cell kinetics can provide insights into the activity of the therapy, appropriate CAR T cell detection methods are essential. Here, we report on the comprehensive validation of a flow cytometric assay for peripheral blood CD19 CAR T cell detection. Further, a retrospective analysis (n = 30) of CAR T cell and B cell levels over time has been performed, and CAR T cell phenotypes have been characterized. Serial dilution experiments demonstrated precise and linear quantification down to 0.05% of T cells or 22 CAR T cell events. The calculated detection limit at 13 events was confirmed with CAR T cell negative control samples. Inter-method comparison with real-time PCR showed appreciable correlation. Stability testing revealed diminished CAR T cell values already one day after sample collection. While we found long-term CAR T cell detectability and B cell aplasia in most patients (12/17), some patients (5/17) experienced B cell recovery. In three of these patients the coexistence of CAR T cells and regenerating B cells was observed. Repeat CAR T cell infusions led to detectable but limited re-expansions. Comparison of CAR T cell subsets with their counterparts among all T cells showed a significantly higher percentage of effector memory T cells and a significantly lower percentage of naïve T cells and T EMRA cells among CAR T cells. In conclusion, flow cytometric CAR T cell detection is a reliable method to monitor CAR T cells if measurements start without delay and sufficient T cell counts are given.

## Introduction

Despite major improvements in the long-term survival of children and young adults diagnosed with acute lymphoblastic leukemia (ALL) (1, 2), the subset of patients with relapsed or refractory (r/r) disease continues to show poor outcomes (3) and new therapeutic approaches are therefore urgently needed. With CD19-targeted chimeric antigen receptor (CAR) T cells, harnessing the cytotoxic potential of T cells and specifically directing it against the malignant cell type, a promising immunotherapy, addressing this need, has emerged (4).

The introduction of second-generation CAR T cells (5, 6) and their success when advancing into clinical trials (7, 8) just one decade ago set the stage for an explosively growing area of research. Since then, large clinical studies were able to provide considerable evidence of the advantages of CAR T cell therapy in r/r pediatric andAlthough not a current standard, the recent best practices of EBMT and JACIE for the management of CAR T cell therapy recommend the monitoring of medium- and long-term CAR T cell persistence (16). Peak expansion levels of tisagenlecleucel in peripheral blood of pediatric ALL patients were shown to be higher in responding patients than in nonresponding patients and were correlated with cytokine levels (17), which in turn are thought to be in causal relationship with the occurrence of adverse events, like cytokine release syndrome (CRS) (18) or immune effector cell-associated neurotoxic syndrome (ICANS) (19). Furthermore, the duration of detectable CAR T cell levels has been found to correlate with event free survival (20). Thus, while the mere monitoring of B cell aplasia (BCA) does also indicate continued functional activity of CAR T cells (21, 22), the direct monitoring of CAR T cell levels provides a deeper dimension of information and can contribute to clinical decision making.

A wide variety of CAR T cell detection methods have been described so far, which were mostly intended for basic science and clinical trial applications. Real-time PCR and flow cytometry have shown the most value for longitudinal monitoring of CAR T cells among them, as they are well established and widely available methods (23–25). Nevertheless, there are still few publications focusing on CAR-T cell monitoring with flow cytometry outside of clinical trials.

In this article, we aim to provide a detailed characterization of the flow cytometric assay which we have been using for the monitoring of CAR T cell levels in patients infused with tisagenlecleucel, including an alternative method comparison with real-time PCR. In addition to that, we analyze the CAR T cell monitoring data regarding detectability and functionality. Finally, we compare the composition of T cell subpopulations between all T cells and CAR positive T cells in several patients.

## Patients And Methods

### Patients and Samples

This study presents data from 29 pediatric and five adult patients, who underwent treatment with tisagenlecleucel (Kymriah^®^) in three European centers after receiving lymphodepleting chemotherapy with cyclophosphamide and fludarabine.

CAR T cell and B cell monitoring was carried out on EDTA-anticoagulated peripheral blood as part of routine follow-up. Additional flow cytometry and real-time PCR tests for analytical validation or subpopulation analysis were performed on leftovers. Cryopreserved aliquots from rinsed tubing of tisagenlecleucel products after infusion were used for spike-in experiments and subpopulation analyses. Leftover samples from randomly selected, anonymous patients who had no history of CAR T cell treatment served as negative controls to determine the assay sensitivity. Our retrospective analysis of CAR T cell and B cell detectability was based on 272 measurements on 30 patients (**Supplementary Table 1**). The study was approved by the local Ethics Committee (#20-807). All CAR T cell patients had given written informed consent.

### Flow Cytometric Detection of CAR T Cells

A four-color flow cytometry panel was designed using a commercial CD19 CAR Detection Reagent (Miltenyi Biotec, Bergisch Gladbach, Germany). The reagent consists of a biotinylated CD19 antigen that specifically binds CD19-targeted CARs. In a second incubation step the biotin-labeled CAR T cells are then stained with a fluorochrome-conjugated anti-biotin antibody.

In brief, 200 µl whole blood were treated for 10 min with 2 ml of NH_4_Cl-based erythrocyte lysing solution (Beckman Coulter, Krefeld, Germany) and washed with PBS, containing 0.5% HSA. After removal of the supernatant down to 200 µl, cells were resuspended and 100 µl were transferred to a new flow cytometry tube. Following 15 min of incubation with 1 µl CD19 CAR Detection Reagent, cells were washed twice and incubated for 15 min with 1 µl Anti-Biotin-PE (Miltenyi Biotec, Bergisch Gladbach, Germany), 10 µl 7-AAD, 5 µl CD3-APC, and 5 µl CD45-KrO (all purchased from Beckman Coulter Immunotech, Marseille, France). After a final washing step, cells were acquired on a NAVIOS flow cytometer (Beckman Coulter, Krefeld, Germany). Cellular debris was excluded based on light scatter properties and CAR T cells were defined as 7-AAD^-^/CD45^+^/mononuclear cells/CD3^+^/CD19 CAR^+^ (**Supplementary Figure 1**).

### B Cell and T Cell Monitoring

B cells, T cells, and other leukocyte subpopulations were regularly assessed on a FC500 flow cytometer (Beckman Coulter, Krefeld, Germany) as described previously (26). In a dual-platform approach, leukocyte counts were measured with the Sysmex XN-1000 hematology analyzer (Sysmex Deutschland, Norderstedt, Germany) and absolute cell counts of leukocyte subpopulations were calculated with the percentages determined by the flow cytometric assay. Relative B cell levels are reported as percentage of the lymphocyte gate.

### Titration of Antibodies for Flow Cytometric Analysis

CAR T cell-specimens were stained with 0.5, 1, 2.5, 5, 7.5, or 10 µl of CD19 CAR Detection Reagent and with the same volume of Anti-Biotin-PE. To ensure comparability, the titration experiments were otherwise conducted concordant with the protocol described above. We determined the optimal antibody volume as the volume with the highest mean fluorescence intensity (MFI) of the positive fraction divided by the MFI of the negative fraction (signal-to-noise ratio).

### Sample Stability Testing

The baseline measurement was performed on the day of sample collection. Subsequently, the specimen was stored at ambient temperature without addition of fixatives or stabilizers and analyzed daily for up to five consecutive days with the CAR T cell detection protocol described above. Sample stability at a particular time point of measurement was assessed on the basis of the percentage of viable leukocytes and the relative percent difference (RPD) of the CAR T cell value from baseline.

### Assay Sensitivity and Specificity

To determine the sensitivity of our assay, cryopreserved cells (in RPMI medium with 20% FCS and 10% DMSO) from rinsed tisagenlecleucel infusion tubing were thawed, washed twice, and spiked into CAR T cell negative whole blood. The spike-in sample was serially diluted in whole blood and measured in triplicates if a sufficient blood volume was available. Un-spiked negative controls were run to assess the mean of blank samples, i.e., the mean event number in the CAR T cell gate. The Limit of Blank (LOB) was calculated as the mean of blank samples + 1.645 standard deviations (SD). Based on this, the Limit of Detection (LOD) was estimated as the LOB + 1.645 SD. Accurate CAR T cell quantification is highly dependent on the number of T cell events acquired in the run. Therefore, LOB and LOD were calculated for CAR T cell event numbers rather than concentrations. The Lower Limit of Quantification (LLOQ) was defined as the lowest concentration above LOD at which the coefficient of variation (CV) of the triplicate measurement achieved < 30% CV (27). Subsequently, the CAR T cell detection protocol was run on leftover CAR T cell negative patient specimens to verify the specificity.

### Precision Measurements

Intra-assay precision was assessed with technical duplicates in a single measurement run on a NAVIOS flow cytometer. Afterwards, the flow cytometry tubes were transferred to a DxFLEX flow cytometer (Beckman Coulter, Krefeld, Germany), and the measurement was repeated on the alternative instrument.

Finally, we established the inter-assay precision on the initial instrument. For this purpose, sample processing and acquisition were performed independently for two more measurement runs. Prior to acquisition, the flow cytometer was powered-down, and daily quality control was repeated. Acceptance criteria were defined as ≤ 25% CV or 30-35% CV at LLOQ, as suggested by Sarikonda et al. (27).

### Real-Time PCR

For the detection of CAR T cells, we established a real-time PCR system. The amplicon is located in the region spanning from CD8 to 4-1BB. This targeted combination is unique for the chimeric construct and minimizes any background signals, which might impact detection of less frequent CAR T-cell levels. After sequencing the CAR from FMC63 IGHV to TCRζ, following primers were designed: forward (CTTCTCCTGTCACTGGTTATCAC), reverse (CTCGTTATAGAGCTGGTTCTGG), and probe (CAAACGGGGCAGAAAGAAACTCCT). Real-time PCR was tested with leftovers from one patient’s infusion bag showing 30% positive cells as confirmed by flow cytometric analysis. Isolated leftover DNA was diluted stepwise in mixed buffy DNA, originating from six CAR T cell negative samples, to simulate lymphocyte background. The determined quantitative range of the assay was 1E-04 (1 positive cell in 10,000 negative cells).

### CAR T Cell Subpopulation Analysis

Immediately after CAR T cell detection with our flow cytometric assay, the sample was reincubated for 15 min with CD62L-FITC, CD45RO-ECD, CD8-APC-A700, CD4-APC-A750, and CD45RA-PacB (all purchased from Beckman Coulter Immunotech, Marseille, France) and acquired on the NAVIOS instrument again. Subpopulations were defined as CD62L^+^ CD45RO^+^ central memory T cells, CD62L^-^ CD45RO^+^ effector memory T cells, CD62L^-^ CD45RA^+^ T EMRA cells, and CD62L^+^ CD45RA^+^ naïve T cells. Stem cell memory T cells are comprised in the naïve population as no discriminating markers were assessed.

### Data Analysis

Dot plots were generated using NAVIOS Software version 1.3 (Beckman Coulter, Krefeld, Germany) or FlowJo Software v10.6.2 (FlowJo, LLC, Ashland, OR). Statistical analyses and graphs were done using Prism 9.0.1. (GraphPad Software, San Diego, CA). Statistically significant differences between subpopulations of T cells and CAR T cells were assessed by two-tailed paired t-tests. As normality testing suggested a departure from normality for the naïve subpopulations, the nonparametric two-tailed paired Wilcoxon signed rank test was chosen for this subset. The Bonferroni-Dunn correction was used to account for multiple comparisons. Adjusted p values were considered statistically significant below 0.05. Spearman’s rank correlation analysis was carried out between flow cytometry and real-time PCR test results.

## Results

### Antibody Titration

**Figure 1:**
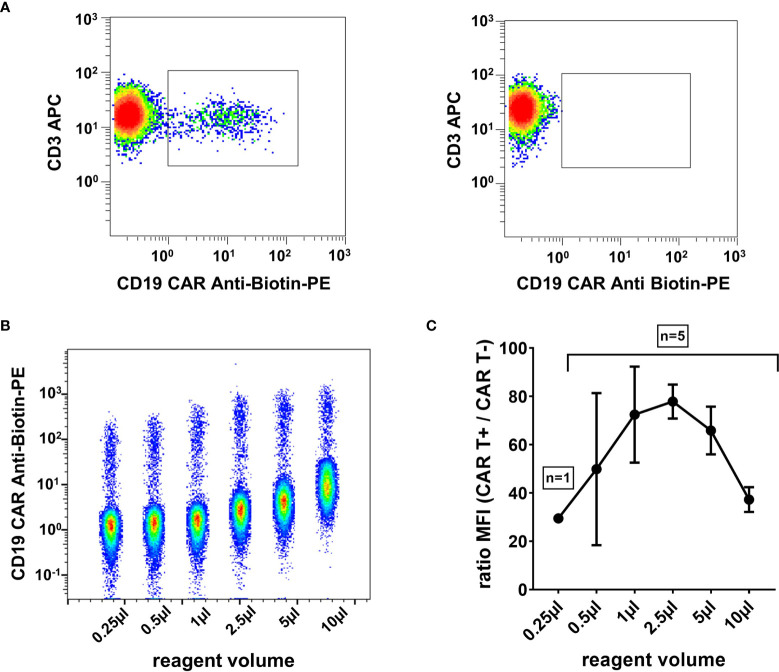
High signal-to-noise ratio with optimally titrated CAR T cell detection reagent. **(A)** Representative flow cytometry plot of a CAR T cell patient sample on day 13 post infusion (left) and a control sample without CAR T cells (right). Plots were gated on viable T cells (7-AAD^-^/CD45^+^/mononuclear cells/CD3^+^). **(B)** Exemplary CAR T cell detection reagent titration series. Six dot plots of flow cytometric measurements with 0.25 µl, 0.5 µl, 1 µl, 2.5 µl, 5 µl, and 10 µl reagent volume, respectively, are depicted in a single concatenated plot. Cells were gated on viable T cells. **(C)** The titration curve, obtained from five independent experiments, shows the maximal signal-to-noise ratio in the range between 1 µl and 5 µl reagent volume. Mean and standard deviation are indicated.

CD19 CAR Detection Reagent and Anti-Biotin PE volumes were titrated to determine the reagent volume that results in optimal separation of CAR positive and CAR negative populations. 1 µl, 2.5 µl, and 5µl were considered within the optimal range (**Figure 1**), and 1 µl was subsequently chosen to be continued with in our CAR T cell monitoring.

### Sample Stability

**Figure 2:**
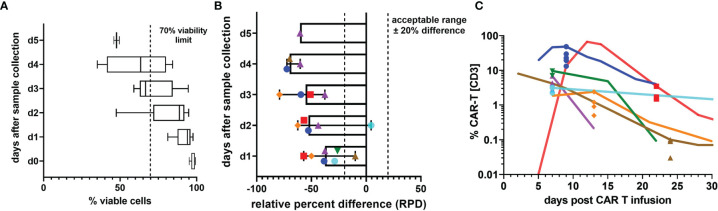
Sample stability. **(A)** Percentage of 7-AAD^-^ viable white blood cells on the day of specimen collection and up to 5 following days. Box plots indicate median, interquartile range, and minimum and maximum. **(B)** The relative percent difference (RPD) of the CAR T cell percentage measured on the day of sample collection compared with the CAR T cell percentages on the following days is shown. Due to limited specimen volumes, the number of days on which stability measurements could be performed varied between 2 and 6 days. Each individual patient sample is represented by one symbol and color. Dashed lines mark the limits of acceptable variation. Median RPDs (columns) and range (error bars) are shown. **(C)** To illustrate the impact of increasing deviations from the d0 value, which is presumed to be the most accurate value, all stability test results are plotted on the respective day of sample collection in the graph and overlayed by the CAR T cell monitoring curves of the patients. The curves run through the d0 value. Colors and symbols match the patients in panel **(B)**.

In clinical routine, patient samples frequently arrive at the laboratory with delay, which makes their timely analysis on the same day difficult. More importantly though, many centers administering CAR T cells have not yet established CAR T cell detection methods, and patient specimens have to be shipped to cooperating centers for analysis. To assess the impact of such measurement delays on cell viability and the reported percentage of CAR T cells, we repeated testing of seven patient specimens at daily intervals. All samples for stability testing were collected within the first month after CAR T cell infusion. While initial leukocyte viability was very high and expectedly declined slowly over time (**Figure 2A**), the RPD of the median CAR T cell value plummeted strikingly early and exceeded our acceptability criterion of 20% difference even on the first day after sample collection (**Figure 2B**). As demonstrated in **Figure 2C**, measurement delays noticeably skewed the result towards lower CAR T cell concentrations. However, neither the general tendency of the course nor the order of magnitude or the detectability were affected.

### Assay Sensitivity and Specificity

**Figure 3:**
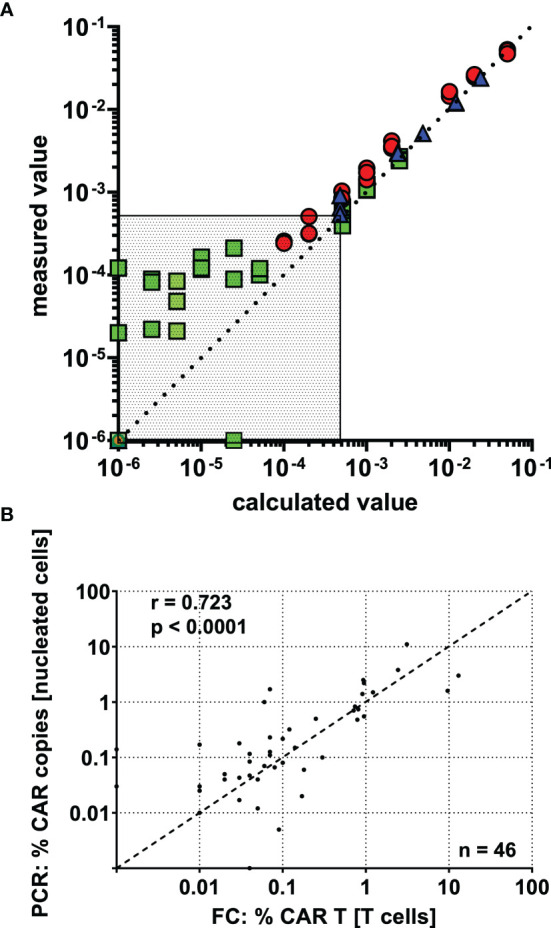
CAR T cell detection shows high precision, linearity, and inter-method agreement down to a lower limit of quantification (LLOQ) of 0.05% CAR T cells. **(A)** CAR-T cells were spiked into CAR T cell negative whole blood, and a dilution series was performed. Based on the percentage of CAR T cells in the undiluted spike-in sample, the “calculated value” was determined by dividing by the dilution factor and plotted against the “measured value”. Data was obtained in three independent experiments (indicated by colors and symbols) with varying amounts of CAR T cells spiked in and different dilution schemes. The region below the assay’s LLOQ is displayed as dotted area. **(B)** 46 post-infusion CAR T cell monitoring samples were concurrently assessed by flow cytometry and real-time PCR. The results show clear inter-method concordance by spearman’s correlation.

To verify the validity of our reported results, we determined a number of assay performance characteristics. First, the LLOQ was established by means of a dilution series in CAR T cell negative whole blood (**Figure 3A**). Results from three independent experiments suggest precise (maximally 27.2% CV at LLOQ) and linear quantification of the CAR T cell fraction down to a LLOQ of 0.05% of T cells, which corresponded to 22 events.

We used negative controls and near-blank dilutions to calculate LOB and LOD at 8 and 13 CAR T cell events, respectively. The mean event number of viable T cells in the parent gate was 45,061. Below the LOD, a departure from linearity was observed, and CAR T cells could not be distinguished from background staining.

Following this, our assay was run on 21 CAR T cell negative patient samples, which were thought to be more comparable with CAR T cell patient specimens than healthy donor blood. Except for one outlier, which showed substantial background staining, all negative samples fell below the detection limit.

### Precision Measurements

We next evaluated the precision of our assay at different levels across the detection range. A high positive (1277 CAR positive events), an intermediate positive (579 CAR positive events), and a low positive (58 CAR positive events) sample were selected, and three different precision parameters were assessed for each of them. The imprecision ranged between 0% and 16.8% CV, with the low positive sample displaying the highest imprecision among them. A fourth sample at the very detection limit (12 CAR positive events) clearly showed more intra-assay (40% CV), inter-assay (32.7% CV), and inter-instrument (20.3% CV) imprecision and thereby exceeded recommended limits for precision.

### Comparison of Flow Cytometry and Real-Time PCR Measurements

46 CAR T cell monitoring samples were independently assessed with both methods, flow cytometry and real-time PCR. Although the methods assay different features of CAR T cells and report in different units, we observed a clear correlation (**Figure 3B**), as shown by Spearman’s correlation coefficient (r = 0.723; p < 0.0001). Of note, the level of agreement between the methods seems to be relatively constant throughout the detection range, supporting acceptable accuracy down to very low frequencies.

### Dynamics of CAR T Cell Kinetics and B Cell Aplasia (BCA)

**Figure 4:**
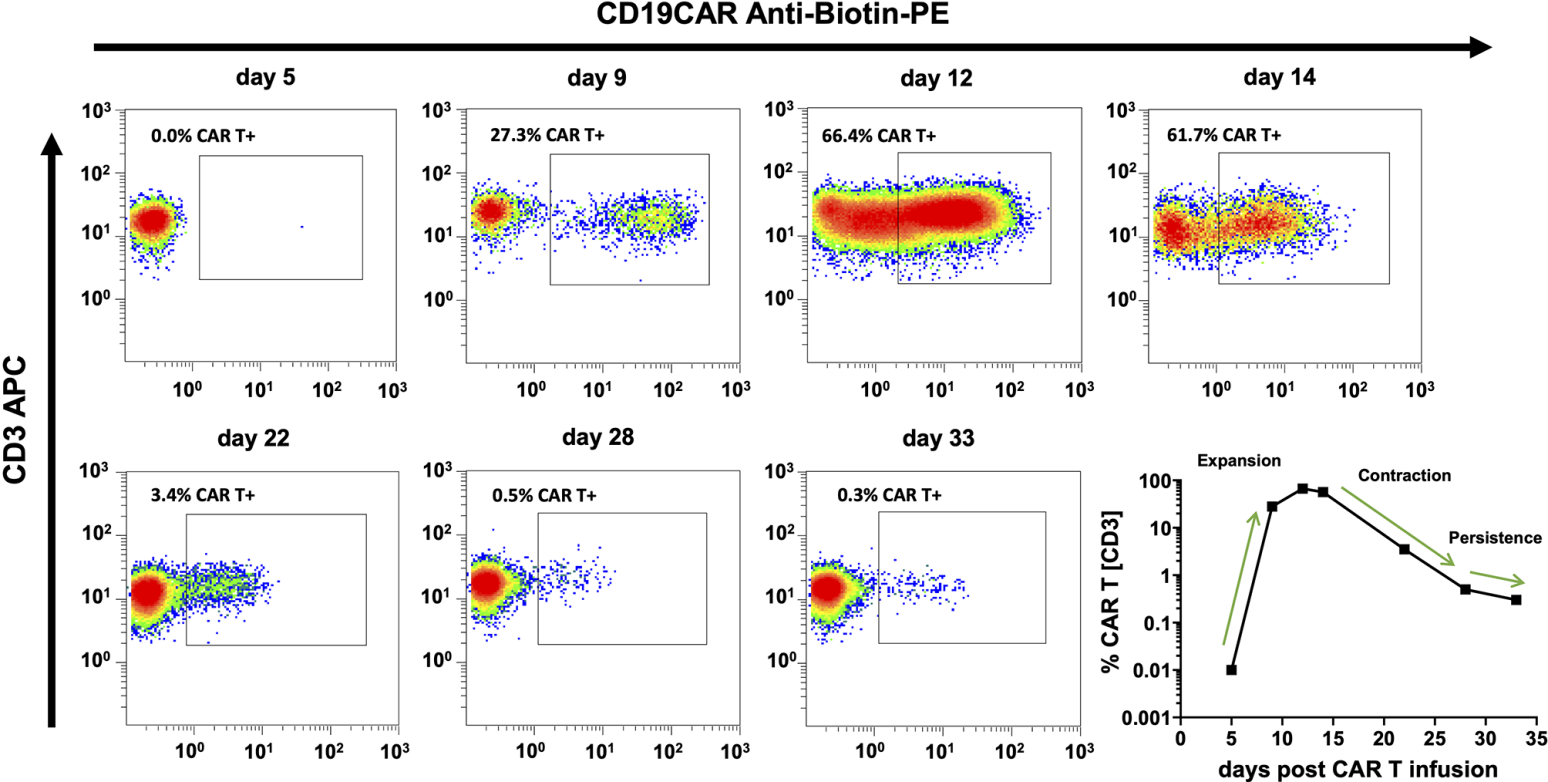
CAR T cell monitoring curves match well with common models of CAR T cell cellular kinetics. CAR T cell cellular kinetics can be broken up into an initial period of exponential growth (expansion), a period of rapidly falling CAR T cell numbers (contraction), and a gradual decline over months or years (persistence). Flow cytometry plots and the bottom right curve depict the CAR T cell monitoring course of the same individual patient. Cellular kinetic phases can be easily identified and are indicated in the bottom right curve.

**Figure 5:**
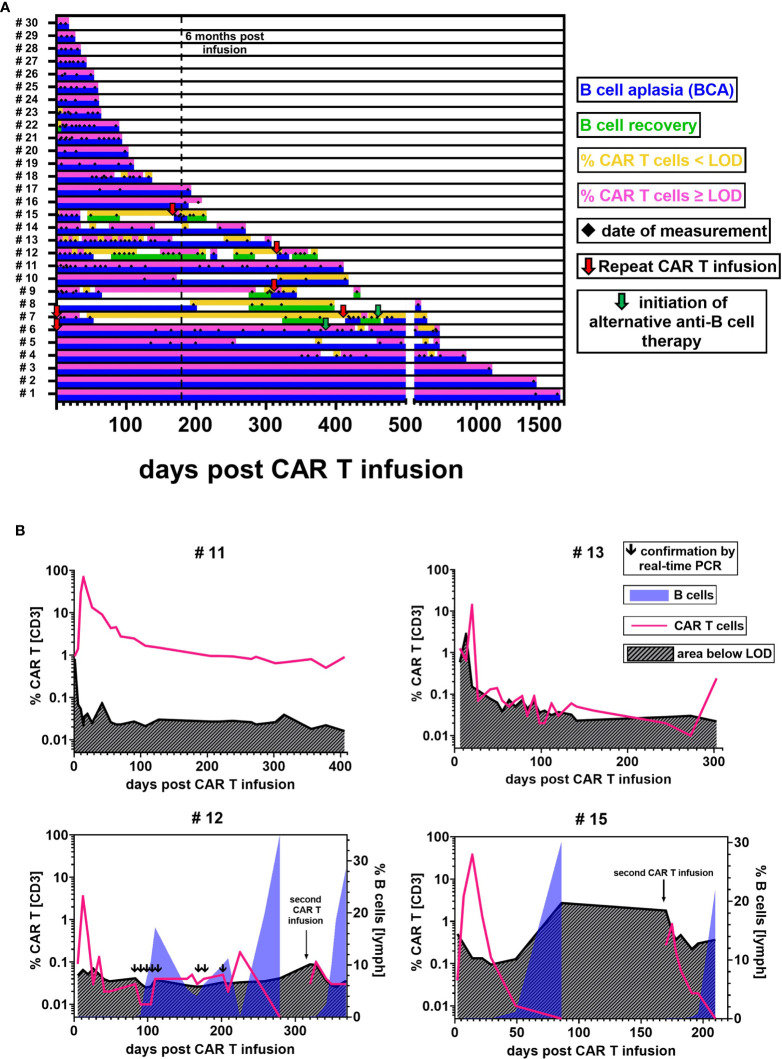
CAR T cell detectability and functional persistence. **(A)** Each lane illustrates the CAR T cell (upper sublane) and B cell (lower sublane) monitoring results for the follow-up of one individual patient. Measurement time points are represented by black diamonds in the center. Periods of CAR T cell frequencies above LOD (pink) and below LOD (yellow) were distinguished to evaluate detectability. Functional CAR T cell persistence was assessed by monitoring B cell levels. Periods with B cells ≤ 0.2% were regarded as BCA (blue). B cell recovery (green) was defined as an increase of the percentage of B cells > 0.2%, starting from the first result with detectable B cells ≥ 0.1%. Red arrows indicate repeat CAR T cell infusions. Green arrows denote the initiation of an alternative anti-B cell therapy. In two patients, day 0 is marked as repeat infusion because our monitoring began at their second infusion. **(B)** Representative curves of CAR T cell monitoring results and their current detection limit as calculated based on our experimentally established LOD of 13 flow cytometric events. Blue overlay areas indicate the recovery of B cells if applicable.

The cellular kinetics of CAR T cells follow uniform patterns after infusion (**Figure 4**). Nevertheless, there is substantial variability between individual patients. Among pediatric ALL patients who received their first CAR T cell infusion, we observed the median peak expansion on day 12 (n = 13; range, 6 to 20) at 37.33% (n = 13; range, 3.53% to 69.36%) CAR T cells. At the last follow-up, 71% (12/17) of patients with more than six months follow-up were in ongoing BCA, which demonstrates functional CAR T cell persistence in the majority of patients (**Figure 5A**).

### Low-Level Persistence Around the Detection Limit

While we confirmed the maintenance of high CAR T cell levels for 1645 and 406 days in two patients (#1, #11) (**Figure 5B** upper left), most patients with BCA for more than 6 months (10/12) exhibited CAR T cell levels around the detection limit (**Figure 5B** upper right). In several of these patients (#4, #5, #6, #10, #13, #14), the CAR T cell percentage fell below and rose above the detectability threshold at different time points. And yet, the ongoing BCA and CAR T cell detectability by real-time PCR in these patients provide evidence of continuous low-level persistence. These inconsistently positive flow cytometric results might in part be explained by variations of the CAR T cell frequency in the patients. Another reason are differences in the T cell count per microliter between samples. If, for example, a sensitivity level of 0.05% is aimed for, a minimum of 26,000 T cell events would be required in the flow cytometric measurement to reach our experimentally established LOD of 13 CAR T cell events. An analysis of the relationship between T cell count and T cell event number showed that most samples with T cells <1000/µl did not meet this sensitivity target, whereas those with T cells ≥1000/µl did (**Supplementary Figure 2**).

### Coexistence of CAR T Cells and B Cells

On the contrary, 29% (5/17) of patients experienced recovery of B cells. To our surprise, we were intermittently able to detect low-level CAR T cell persistence during B cell recovery up to 18.6% in three patients (#7, #9, #12). In patient #12, the coexistence of CAR T cells and B cells was repeatedly confirmed by real-time PCR testing.

Interestingly, while CAR T cell concentrations were almost always ≤ 0.05% in samples showing B cell recovery, we found that a transient reoccurrence of BCA in two patients (#8, #12) after prolonged periods of B cell recovery was accompanied by an increase of CAR T cell levels to ca. 0.2% (**Figure 5B** lower left).

### Repeat CAR T Cell Infusions

Among five patients who received more than one CAR T cell infusion, one patient (#6) achieved durable CAR T cell persistence above the detection limit with BCA lasting for one year before the patient was given other anti-B cell therapies for CD19 negative relapse. A second and a third infusion in patient #7 and a second infusion in patients #9, #12, and #15 though, showed limited re-expansion with a CAR T cell peak at 12.3%, 0.12%, 1.97%, 0.10%, and 0.85%, respectively. Additionally, CAR T cell levels declined rapidly below the detection limit. We observed B cell recovery 328 and 29 days after the second and third infusion in patient #7 and 118, 27, and 23 days after the second infusion in patients #9, #12, and #15 (**Figure 5B** lower right).

### CAR T Cell Subpopulations During Initial Expansion

**Figure 6:**
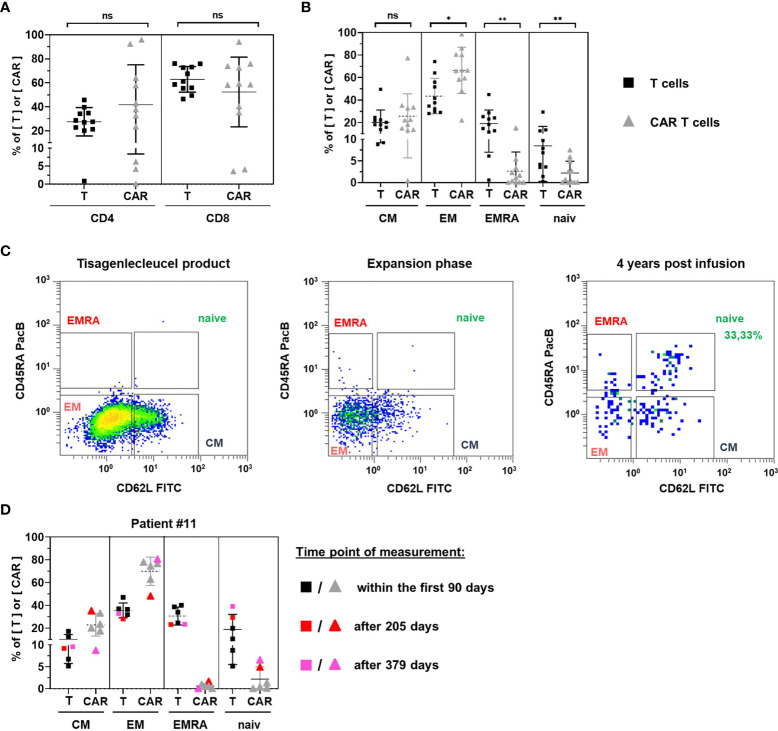
Phenotypic analysis. CD4 and CD8 positive subsets **(A)** and subsets positive for central memory, effector memory, EMRA, and naïve marker signatures **(B)** were compared between T cells (black squares) and CAR T cells (grey triangles) for 11 patients in their initial expansion phase. **(C)** Representative gating of naïve CAR T cells in subpopulation analyses of tisagenlecleucel product cells (left) and patient samples during the period of exponential expansion (middle) as compared to one patient’s sample 4 years after CAR T cell infusion (right). **(D)** subpopulation analysis was performed four times within the first 90 days (uncolored symbols) and once 205 days (red symbols) and 379 days (pink symbols) post CAR T cell infusion of patient #11. Horizontal bars represent the mean. Vertical error bars indicate the standard deviation. ns, not significant; *p < 0.05; **p < 0.01.

The phenotypic composition of 11 CAR T cell monitoring samples from seven pediatric ALL patients and four adult NHL patients in their initial expansion phase was evaluated. The mean proportion of CD4 or CD8 CAR T cells and CD4 or CD8 T cells did not differ significantly (**Figure 6A**). We observed an inversed CD4/CD8 ratio, which was 0.44 (27.53%/62.92%) for all T cells and 0.79 (41.72%/52.4%) for CAR T cells. Yet, there was large variation between individual patients and the CAR T cells of two of them were almost completely composed of CD4 CAR T cells with CD4/CD8 ratios above 20.

Comparison of CAR T cell subsets with their T cell counterparts showed a significantly higher frequency of highly differentiated effector memory T cells (p = 0.0346) and a significantly lower frequency of less differentiated naïve T cells (p = 0.0039) among CAR T cells (**Figure 6B**). In addition, the terminally differentiated T EMRA subset was significantly decreased (p = 0.0063).

Among CAR T cells, effector memory (66.39%) and central memory (25.69%) subsets accounted for the vast majority of cells. Naïve CAR T cells (2.20%) and CAR T EMRA cells (2.62%) were only detectable in low frequencies.

### Naïve CAR T Cell Fraction May Increase During the Persistence Period

To resolve rare subsets, the phenotypic analysis of circulating CAR T cells requires high CAR T cell numbers. Consequently, most patient samples in the persistence phase of CAR T cells are not evaluable as they tend to be at the limit of detection. One patient (#1), however, presented 4 years after CAR T cell infusion with a frequency of 0.74% CAR T cells, allowing us to perform a subpopulation analysis. In contrast to the CAR T cell composition in tisagenlecleucel infusion bags or during the initial expansion period, we found 33.33% naïve CAR T cells in this patient (**Figure 6C**).

The notion of increased proportions of naïve cells during CAR T cell persistence was corroborated by the longitudinal follow up of another patient who maintained high CAR T cell numbers 27, 55, 63, 70, 205 and 379 days after CAR T cell infusion (**Figure 6D**). While naïve CAR T cells ranged between 0% and 1.29% within the first 90 days, they represented 4.88% and 6.52% of CAR T cells on days 205 and 379 post infusion, respectively. The rise of the naïve CAR T cell fraction was accompanied by an increase of naïve T cells among all T cells as well as a continuous decline of the overall CAR T cell frequency among peripheral blood T cells.

## Discussion

Miltenyi’s CD19 CAR Detection Reagent has previously been used by other European groups to monitor CAR T cell kinetics after infusion (28–31). This is the first publication, however, that presents a comprehensive analytical validation of a CAR T cell detection assay with the reagent. We determined the optimal reagent volume for our assay to range between 1 µl and 5 µl and found evidence for the instability of CAR T cell levels over time. Ideally, the measurement should therefore be performed on the day of specimen collection.

Due to their large and activated morphology, CAR T cells can resemble monocytes. To ensure that all CAR T cells are included in the analysis, we therefore recommend setting broad mononuclear gates rather than narrow lymphocyte gates. Subsequently, monocytes should be excluded based on specific surface markers (e.g., CD3, CD14).

Our serial dilution experiments showed, even at high dilutions, linear and precise CAR T cell quantification. The LOD was calculated to be at 13 CAR T cell events and high specificity > 95% was confirmed by testing 21 negative control samples.

Further assay performance testing demonstrated excellent precision at high, intermediate, and low CAR T cell concentrations. The quantification of CAR T cell levels at the LOD, however, failed to reach the acceptance criteria proposed by Sarikonda et al. Considering the high statistical variation that is inherent in rare event detection with very low event numbers, a high uncertainty must be expected (32, 33). Thus, we recommend thinking of results between LLOQ and LOD merely as positive results without reliable quantitative information.

We chose a low input blood volume of 200 µl to save patient blood and reduce reagent costs, thereby facilitating regular CAR T cell measurements to be part of the routine monitoring after CAR T cell infusions in the pediatric setting. The analytical sensitivity of our assay with a LLOQ at 0.05% compares well with other flow cytometric CAR T cell detection assays (22, 30, 34). The patient’s T cell count, however, remains a limiting factor, and T cell count variations translate *via* the number of T cell events into an altered LOD and LLOQ. In our experience, the sensitivity was most likely to be insufficient in samples with a T cell count <1000/µl. Thus, it might be advisable in such samples to raise sensitivity by stepping up the input volume, thereby concentrating more cells and achieving higher event numbers. Badbaran et al. applied the CD19 CAR Detection Reagent with an input blood volume of 1 ml, which allowed for the acquisition of high event numbers in all specimens (28).

Finally, the accuracy of our measurement results is evidenced by the correlation of real-time PCR and flow cytometry methods. Both approaches are widely used for the detection of CAR T cells and complement each other. Flow cytometry is faster and, as it can assess numerous parameters in parallel, monitoring of CAR T cells can be combined with other immune cell populations. Likewise, different subsets within the CAR T cell population can be distinguished. Due to its superior sensitivity, real-time PCR might be used to confirm uncertain flow cytometric results or to quantify CAR T cells in paucicellular samples, such as cerebrospinal fluid.

The second part of this article is about the retrospective analysis of the CAR T cell monitoring data of 30 evaluable patients with a total of 272 measurements. Basic parameters of CAR T cell kinetics and pharmacodynamics, such as the median peak expansion and the proportion of patients with durable BCA, are well in line with the results of previous studies on tisagenlecleucel (20, 22).

Since we are performing our monitoring for only one and a half years so far and sample selection was based on availability in the laboratory, the inclusion of patients whose CAR T cell infusion dates back several years might be biased towards patients with long-lasting response, who did not die or pursue alternative treatments. Nevertheless, these patients demonstrate the ability of tisagenlecleucel to persist for years and the suitability of our assay to detect it.

More importantly, even though the persistence of CAR T cells and BCA are established favorable prognostic factors, they do not preclude events such as CD19 negative or extramedullary relapses, and our analysis therefore makes no claims about clinical outcome.

Following the phases of multi-log expansion and rapid contraction, CAR T cells enter a phase of gradual decline. While this final phase took place at remarkably high levels in some individual patients, most patients maintained very low levels, at times even below the detection limit. Although the ongoing BCA in most patients demonstrates that such low concentrations can be sufficient to maintain efficient CAR T cell functioning, it might be that falling below an individual threshold level contributes to reduced CD19-specific CAR T cell activity, as might be exemplified in the five patients with B cell recovery, who had no or noticeably low CAR T cell levels detectable.

Hence, functional persistence of CAR T cells has to be distinguished from persistence in the sense of existence. We repeatedly confirmed the simultaneous circulation of B cells and CAR T cells. Additionally, the observation of reoccurrence of BCA concomitant with an increase of CAR T cell levels raises the question of whether and under what conditions the inactive state of CAR T cells can be reversed. Concepts like vaccines prompting the remaining CAR T cells to re-expand to sufficient concentration (35) may therefore merit close attention.

The CAR T cell monitoring curves of repeat treatments with tisagenlecleucel appear to differ markedly from curves of first treatments. Only one patient achieved durable re-engraftment of CAR T cells, as indicated by ongoing CAR T cell detection and long-lasting BCA. Although we observed the re-expansion of CAR T cells and the transient occurrence of BCA in all other patients too, the peak expansions were low and the recovery of B cells early. Gauthier et al. recently reported similar observations of CAR T cell and B cell kinetics after repeat CAR T cell infusions in a large cohort of 44 adult patients with different B cell malignancies. Interestingly, they found an association between ALL and lower CAR T cell expansions compared to other B cell malignancies (36).

In the third part of this study, we aimed at shedding light on the phenotypic composition of CAR T cells once they expand in the recipient’s body. Significantly higher frequencies of effector memory T cells and significantly lower frequencies of naïve T cells among CAR T cells are consistent with progressive T cell differentiation after extensive target-antigen exposure and proliferation. Moreover, higher frequencies of naïve CAR T cells were observed during the persistence phase. One possible explanation might be that, while memory and effector T cells die off gradually, the proportion of naïve (or stem cell memory) T cells may increase and may play a role in sustaining a long-lived CAR T cell memory pool. Singh et al. demonstrated that naïve T cells primarily give rise to stem cell memory and central memory T cells during *in vitro* expansion (37), which in turn have been shown in other studies to be associated with enhanced *in vivo* persistence (38, 39). Additionally, stem cell memory CAR T cells were found to confer long-lasting anti-leukemic responses in xenograft models (40) as well as in humans (41). Due to the lack of discriminating markers, however, we are unable to distinguish stem cell memory T cells from naïve T cells and cannot determine to which extend they contributed to the CAR T cell pool.

In conclusion, we report on the analytical validation of a flow cytometric assay for the detection of CD19-specific CAR T cells and the analysis of CAR T cell monitoring data generated by its use. Precise quantification was demonstrated down to 22 events or 0.05% of T cells and specific detection down to 13 events. A correlation with real-time PCR results was found, and the advantage of early sample measurement due to reduced stability was pointed out. Further, the cellular kinetics following tisagenlecleucel treatment were analyzed, and the coexistence of CAR T cells and B cells in certain patients was confirmed. Finally, the analysis of CAR T cell subpopulations at peak expansion found the effector memory subset to be significantly higher and naïve and T EMRA subsets to be significantly lower.

## Data Availability Statement

The original contributions presented in the study are included in the article/**Supplementary Material**. Further inquiries can be directed to the corresponding author.

## Ethics Statement

The studies involving human participants were reviewed and approved by the medical ethics committee of the University Hospital Frankfurt. Written informed consent to participate in this study was provided by the participants’ legal guardian/next of kin.

## Author Contributions

AP, MB, HK, CC, and SH conceived and designed the experiments. AP, HK, JBD, and SE performed the experiments. AP, MB, HK, and SH analyzed the data. MB, CC, ER, AJ, SH, and PB coordinated the research. AP, MB, HK, and SH contributed to reagents, materials, and analysis tools. ER, AJ, RM, PGS, OB, GB, and PB contributed to patient and sample recruitment. AP wrote the paper. JHK, TK, SH, and PB supervised the research. All authors revised the manuscript and approved the final version of the manuscript.

## Funding

This project was supported by “Frankfurter Stiftung für krebskranke Kinder”, “Hilfe für krebskranke Kinder Frankfurt e.V.”, and “Frankfurter Promotionsförderung Promotionsstipendium” (F54/2020; R254/2020). The funders had no role in study design, data collection and analysis, decision to publish, or preparation of the manuscript.

## Conflict of Interest

PB declares research grants from Neovii, Riemser, Medac (to Institution); advisory board for Novartis, Cellgene, Amgen, Medac, Servier (personal and to Institution); Speakers Bureau of Miltenyi, Jazz, Riemser, Novartis, Amgen (to Institution), and patent and royalties from Medac.

The remaining authors declare that the research was conducted in the absence of any commercial or financial relationships that could be construed as a potential conflict of interest.

## Publisher’s Note

All claims expressed in this article are solely those of the authors and do not necessarily represent those of their affiliated organizations, or those of the publisher, the editors and the reviewers. Any product that may be evaluated in this article, or claim that may be made by its manufacturer, is not guaranteed or endorsed by the publisher.
