# Supplementary figures and images for: Monitoring of Circulating CAR T Cells: Validation of a Flow Cytometric Assay, Cellular Kinetics, and Phenotype Analysis Following Tisagenlecleucel
Source: Front Immunol. 2022 Mar 2;13:830773. doi: 10.3389/fimmu.2022.830773 (PMC8926389; doi:10.3389/fimmu.2022.830773)

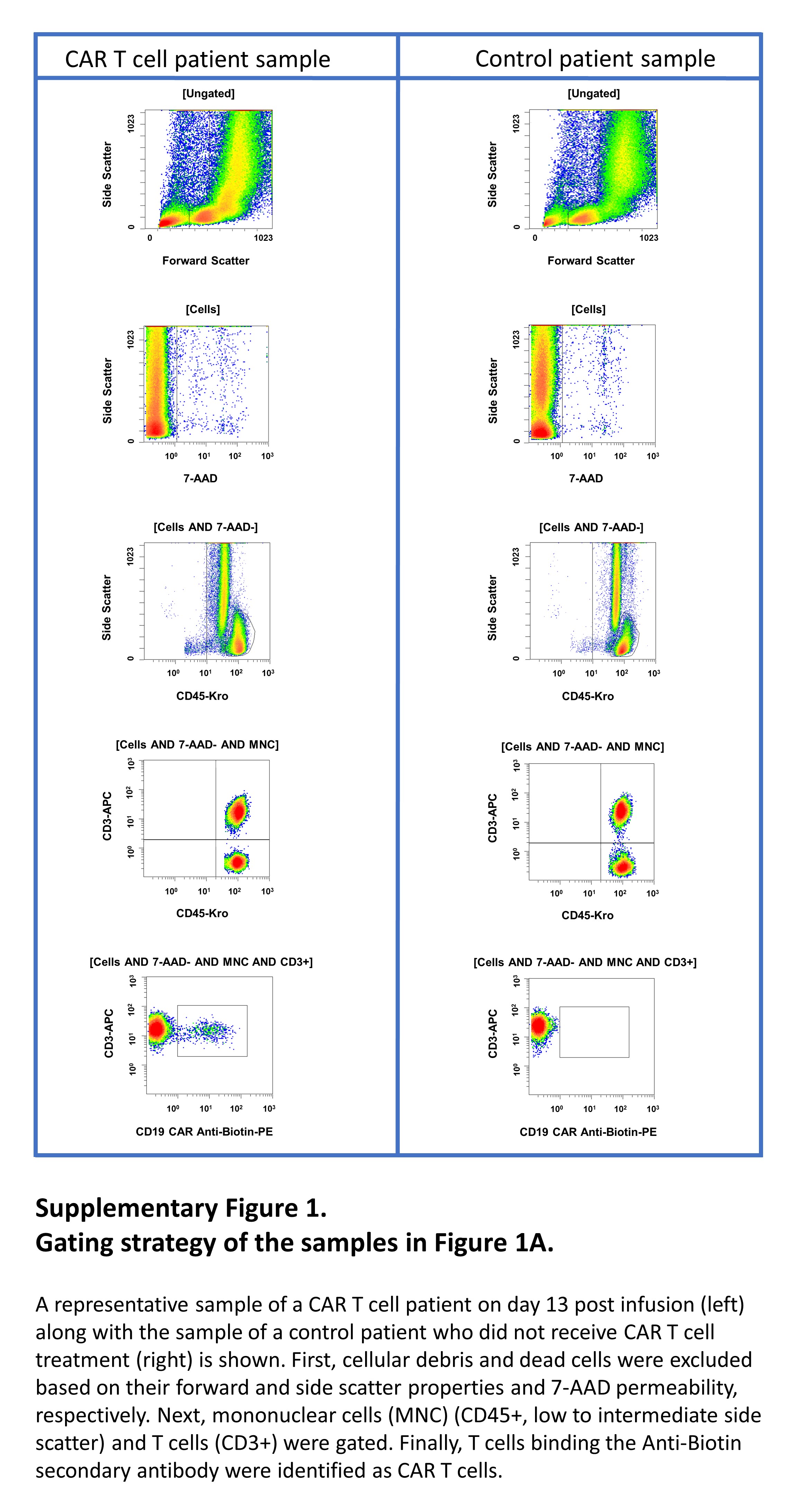

Supplement: Supplementary file 1 [file Image_1.jpg]

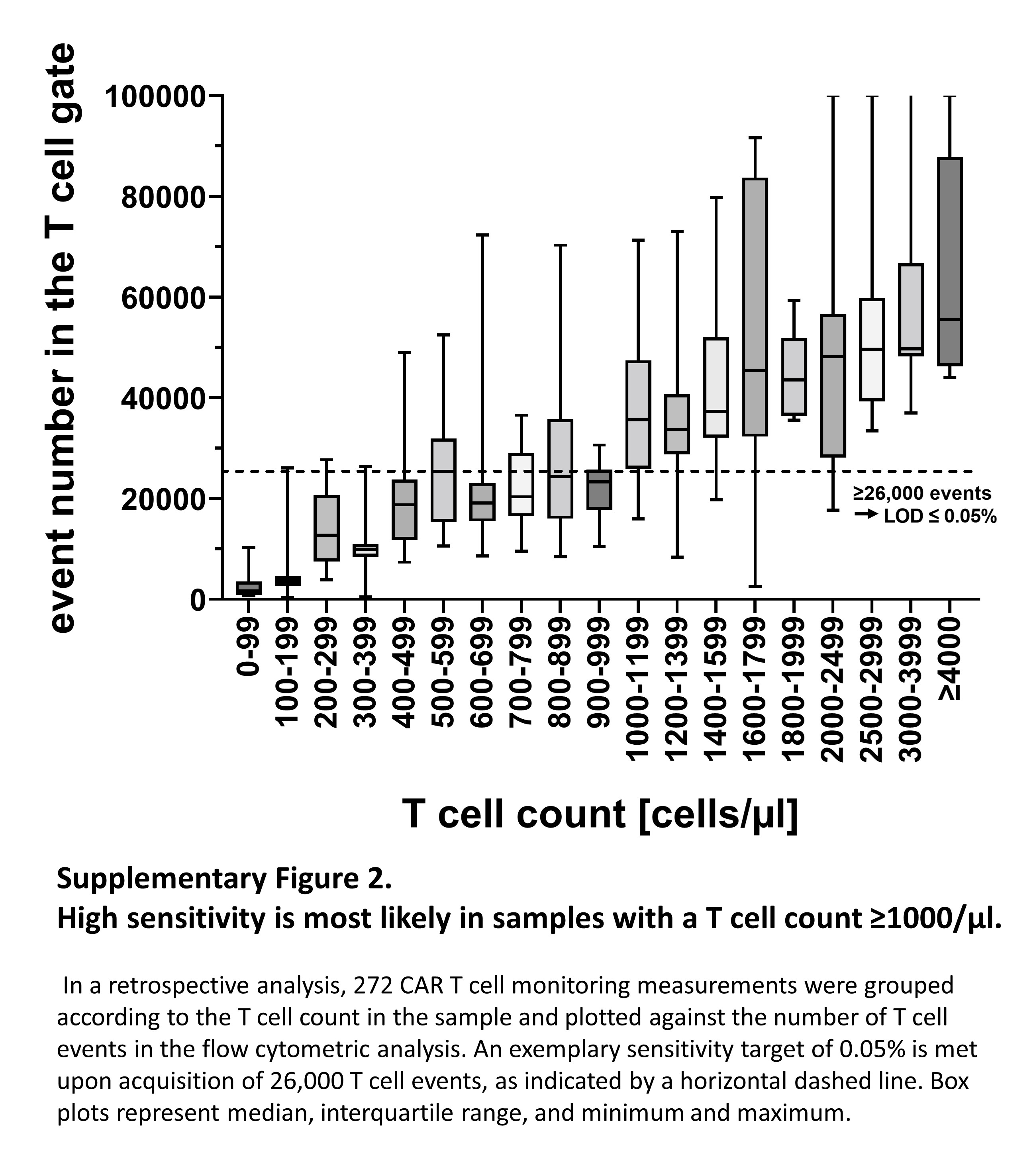

Supplement: Supplementary file 2 [file Image_2.jpg]
